# Anatomy and Physiology

**Published:** 1837-10

**Authors:** 


### PART THIRD.

### Selections from the Foreign Journals.

---

### ANATOMY AND PHYSIOLOGY.

### *On the Structure of the Retina in Man, and the Mammalia generally.* By Dr. C. M. Gottsche, of Altona.

About two years and a half since, Dr. Gottsche made, in Copenhagen, the interesting discovery that a distinct nervous radiation might be seen in the retina of the calf; and, after having shown, by repeated examination in different individuals of the vertebrate classes, that the appearance was constant, it was lastly sought for in the human eye, and discovered.

Authors generally describe that part of the retina which they conceive to be the expansion of the optic nerve as a *medullary expansion*, but the filamentous structure in man is denied by all. Such is the account, at least, given by Meckel, Burdach, Hildebrandt, Weber, &c. Amongst the mammalia, Arnold has found the fibrillæ in the hare and pig alone, but denies their existence in the calf, &c. This description of the distribution of the optic nerve would make it an exception to what is considered an essential characteristic of the terminations of the other nerves of the senses. It is not practicable, in the fresh eyes of mammalia, to show this filamentous structure by simple dissection; therefore, Dr. Gottsche had recourse to the assistance afforded by chemical reagents; and the chief secret consisted in softening and removing the compact layer which lay beneath the nervous fasciculi. Maceration appeared to be a means adapted to effect this, but by it the fibrillæ are only preserved immediately around the circumference of the optic nerve, or at best as far as the middle of the retina. Subsequently, solutions of carbonate and nitrate of potassa were employed; but nothing appeared to answer the purpose so well as a solution of one part of corrosive sublimate in three parts of sulphuric æther. After a sufficient maceration, ten minutes' manipulation with a camel-hair pencil and the above solution are sufficient to produce a most perfect and beautiful preparation of the radiation of the ultimate filaments of the optic nerve.

An eye being procured, as fresh as may be, all the external tunics, including the choroid, are removed; a narrow rim of sclerotic immediately surrounding the optic nerve being alone left. The retina is now loosened from the hyaloid membrane, and spread out with its *inner* surface upon a piece of black paper, or, which is better, upon a piece of glass painted black on one side: it will be found necessary to snip the retina at one part of its circumference, in order that it may lie smooth and flat. If the eye be that of a recently killed animal, it is advantageous to macerate the retina, thus prepared, for a time in water. The period of maceration must be decided by the temperature of the weather, and varies from one day to eight: the requisite amount of softening may be ascertained by means of a camel-hair pencil. As soon as the compact layer above mentioned can be wiped away in small fragments from around the optic nerve, the preparation may then be proceeded with. The retina being now spread out so as to lie perfectly smooth, a few drops of the sublimate solution are allowed to spread themselves over its surface, which is at the same time gently brushed with the pencil: by a repetition of this process, the compact layer yields, and the small flakes are washed away by dropping water or alcohol on the surface. If the preparation be now macerated in spirit for a week, and again cleansed, it is fitted for microscopical examination.

If a fresh eye be treated in the above manner, it is rendered unfit for the desired object by the hardening of the dense layer already spoken of, and the increased firmness of connexion between it and the nervous fibrils.

*Pfaff's Practische und Kritische Mittheilungen, &c.* Heft xxxiv. 1836.

---

*Microscopical Observations on the visible Motion of the Globules of Lymph in the Lymphatics of Tadpoles.* By Professor E. H. Weber, of Leipzig.

It is already known, from Panizza's beautiful investigations, that, in amphibia, many lymphatics lie in the cavity of other larger lymphatics, and that the coats of the former are thus on every side washed by the contents of the latter. This observation Professor Weber and his brother found confirmed on examination of the vascular system of the Python tigris: most of the blood-vessels, indeed, do not lie in the cavity of a single larger vessel, but they are surrounded by a compact net of lymphatics in such a manner that the coats of the central vessel are still washed by the lymph contained in the others; and the only difference lies in the space surrounding the blood-vessels being divided by threads and thin partitions into several communicating compartments.

On selecting a transparent spot on the surface of the tail of the tadpole, it is easy, with the aid of a powerful microscope, to perceive the motions of the globules of blood in the veins; and attentive observation will point out the movement of the globules of lymph in the concomitant lymphatics. When thus viewed, many blood-vessels appear to be broader than the stream of blood, and the observer distinguishes, on both sides of the vessel, a transparent border, into which no globules penetrate. This border might be taken for the lateral parts of the cavity of the blood-vessel, but more attentive observation shows that it must be separated from this cavity, although no partition is visible. The flattened oval globules of the blood are never seen to enter these lateral compartments, but from time to time a round lymphatic globule passes along them. These globules of lymph move very slowly, and often stand still or even retrograde; whilst the globules of blood move along with considerable rapidity, and Weber infers that they are separated from each other by a partition,—first, because none of the globules of the blood are ever observed in the lateral compartments; and, secondly, on account of the great difference of progressive motion of the two varieties of globules. But the space in which these lymphatic globules move extends around the blood-vessel, and, if the microscope be so adjusted as to make the distinct field of vision fall behind the stream of blood, the lymphatic globules, moving slowly along, may be perceived behind the stream. It sometimes happens that blood-vessels are met with which have no apparent transparent border; but this is not evidence sufficient to prove that it does not exist, as the refraction of the light may be such as to prevent it from being visible. Weber found the lymphatic globules to vary in size from 0.003 to 0.00519 of a Parisian line. We are possessed of no means of fixing positively their rate of motion, but it seemed to Weber to be at least ten or twenty times slower than that of the blood. Several other authors have remarked the transparent border or edge of the blood-vessels, and have attributed it to various causes. Blainville supposed that the appearance was owing to the inner coat of the blood-vessel being lined, as it were, with a coating of serum.

*Müller's Archiv. Jahrgang*, 1837. Heft ii.

---

*Case of Loss of Power of Volition over some of the Cerebral Nerves.* By Dr. Magnus, of Berlin.

A young married woman, æt. 25, lost her husband before the birth of her first child, and the latter died soon after birth. During her recovery, the lochial discharge was suddenly suppressed in consequence of a violent fit of anger, which was followed by hemiplegia and symptoms of insanity. The case was viewed as apoplexy by her medical attendant, and, under antiphlogistic treatment, she gradually recovered. In the month of October, 1835, she was exposed to cold during the flow of the menses, which were in consequence partially suppressed, and during the next four weeks she continued to complain, without, however, being able to specify precisely what ailed her. At her next period the menses did not appear, but she had an attack similar to that under which she had suffered in childbed. The power of speech was totally lost, but the hemiplegia was not so complete as formerly. She was treated on the same principles, and in some measure with success: the weakness in the extremities was almost completely removed, but the power of speech did not return.

Dr. Magnus was called to attend her in June, 1836. The patient's face was perfectly smooth, without the slightest wrinkle, and totally devoid of expression. She was unable to retain the saliva, which flowed through the half-open mouth; speech was completely lost, but the patient could produce an inarticulate sound, without, however, having any power of modifying it. She had no command over the voluntary muscles of the face; she could not contract the brows nor elevate the alæ nasæ; but the eyelids, although resisting the influence of the will, closed involuntarily on the sudden approach of a body towards the eye, or on the application of a strong light; the iris retained its motions in full integrity. The patient possessed the power of motion of the lower jaw, but its movements were slower and less energetic than in the normal state. The tongue did not obey the will; the patient could neither protrude it from the mouth, nor move it within its cavity. In mastication, she was obliged to push the bolus from side to side with the finger, and then to push it backwards till it came within the action of the involuntary muscles, when all the muscular motions connected with deglutition followed exactly as in the healthy state. The sense of taste remained unimpaired. The muscles of the face, although insensible to volition, were affected by some states of the mind. The patient smiled and laughed, and the muscular motions thus produced were exactly the same as those observed in the normal condition. The sound of her laughter was different from the inarticulate tones she at other times produced, and, although partaking somewhat of the nature of a grunt, it underwent modification, showing that the muscles of the larynx, although beyond the reach of volition, were capable of being affected by internal stimulus.

The case is important, as proving that the closing of the eyelids, on the sudden approach of a foreign body, is involuntary; but, perhaps, the most remarkable feature of the case consists in the power of smiling and laughing, which the patient retained, notwithstanding the complete loss of voluntary power over those muscles which are subservient to the expression of these emotions. This phenomenon admits of two modes of explanation: first, the stimulus which produces in the brain the conception of the laughable may pass directly to the roots of the nerves of motion, without affecting in its passage the organ of the will. But, as this theory, according to Dr. Magnus, would suppose a distinct organ of the will, which, in the present state of our knowledge, cannot be recognized, he adopts the second theory, which supposes that, either by compression or some other cause, the texture of the nerves had undergone a change, by which their conducting powers were so altered that, whilst they refused to convey the stimulus of the will, they nevertheless remained obedient to the involuntary stimulus of the conception of the ridiculous.

*Müller's Archiv. fur Anatomie, &c. Jahrgang,* 1837. Heft ii.

